# Determination of prednisolone concentration in human breast milk and plasma of breastfed infants: study protocol of a Swedish multicentre low-intervention clinical trial

**DOI:** 10.1136/bmjopen-2024-097898

**Published:** 2025-05-22

**Authors:** Jenny Svedenkrans, Karin Hellgren, Karin Backlund, Nina Perrin, Erika Timby, Karin Nilsson, Jennifer Drevin, Erica Sundell, Laura Shaughnessy, Mats Hansson

**Affiliations:** 1 Department of Clinical Science, Intervention and Technology (CLINTEC), Division of Pediatrics, Karolinska Institutet, Stockholm, Sweden; 2 Department of Neonatalogy, Karolinska Hospital, Stockholm, Sweden; 3 Division of Clinical Epidemiology, Department of Medicine, Karolinska Institutet, Stockholm, Sweden; 4 Centre for Rheumatology, Academic Specialist Centre, Stockholm Health Services, Stockholm, Sweden; 5 Södra Älvsborgs Hospital Borås, Borås, Sweden; 6 Center for Pediatric Clinical Studies, Astrid Lindgren Children’s Hospital, Karolinska University Hospital, Stockholm, Sweden; 7 Department of Clinical Sciences, Umeå universitet Medicinska fakulteten, Umeå, Sweden; 8 Centre for Research Ethics and Bioethics, Department of Public Health and Caring Sciences, Uppsala University, Uppsala, Sweden; 9 UCB Pharma, Morrisville, North Carolina, USA

**Keywords:** RHEUMATOLOGY, Maternal medicine, Medicine, Reproductive medicine

## Abstract

**Introduction:**

Many women need to use medications during breastfeeding. Very few medications have been adequately monitored, tested and labelled with safety information for this use. Prednisolone is one of these drugs. We aim to conduct a multicentre low-intervention clinical trial to determine the concentration of prednisolone in plasma of breastfed infants of lactating women treated with prednisolone. In addition, we will measure the concentration in maternal plasma and breast milk and calculate the daily infant dose (DID) and relative infant dose (RID). Infant cortisol levels will be analysed as a measure of clinical effects in the infants.

**Methods and analysis:**

The study will be conducted at departments of obstetrics and gynaecology and specialist maternity and paediatric outpatient clinics in Sweden. We aim to include 30 lactating women treated with prednisolone and their breastfed infants. Breast milk and blood will be collected merely to study the secretion of prednisolone into breast milk and transfer to the infant. Participants will be treated with prednisolone according to their physician’s prescription. Study visits take place when the infant is approximately 6–8 weeks old. Milk and blood sampling of the mother will be performed at 1 hour after drug intake, in conjunction with the infant being fed. Blood sampling of the infant will be performed 2 hours after the feed. Breast milk and plasma will be biobanked for future research. Recruitment was initiated in 2024 and is ongoing. Patient representatives from the Swedish Rheumatism Association were involved in the planning of the study, and the organisation is providing information about the study on their website.

**Ethics and dissemination:**

The clinical trial was approved by the Swedish Medical Product Agency (Dnr. 5.1.1-2023-104170). The results will be published in peer-reviewed scientific journals and disseminated at scientific meetings and through patient organisations’ websites.

**Trial registration number:**

The clinical trial protocol is available via the Clinical Trial Information System at the European Medicines Agency (No. 2023-508913-18-00). It is also registered and publicly accessible at the EU PAS Register (EUPAS 1000000059).

**STRENGTHS AND LIMITATIONS OF THIS STUDY**

- The main strength of the study is that the amount of prednisolone transferred through the breast milk will not just be estimated but measured in the infant’s plasma.
- All procedures for collection, storage and analyses adhere to regulatory standards and recommendations from the Food and Drug Administration regarding lactation studies in order to obtain information fit for label.
- By obtaining informed consent both from the woman and her partner for biobanking and future research, there is a possibility to perform yet unspecified, but ethically approved studies in the future, without additional sampling.
- A limitation of the study is that it is only conducted in Sweden, thereby limiting the relevance for other countries where prescription patterns may be different.
- It is a limitation that sampling is performed as a single measurement, making calculations on infants’ total drug intake less reliable.

## Introduction

 More than 5 million women get pregnant in the European Union (EU) every year, and a majority take at least one medication during pregnancy and breastfeeding.^1^ However, very few medications have been adequately monitored, tested and labelled with safety information for use in pregnant and breastfeeding women.^2 3^ The field, while inherently difficult to study, has suffered from a lack of systematically gathered insights that could lead to more effective data generation methodologies. Fragmentation and misinformation result in confusing and contradictory communication and perception of risks by both health professionals, breastfeeding women and their families.

In clinical settings, the issue of medical safety while breastfeeding often surfaces. Without sufficient evidence of safety, it’s tempting to take a precautionary approach by discontinuing the prescription during breastfeeding or recommending abstaining from breastfeeding. There is a cost to this approach, both for the woman and for her child. The woman may not receive adequate medical treatment, or the woman and her child miss out on the well-known positive effects of breastfeeding.^4^ The lack of evidence regarding the transfer of drugs to the breastfeeding infant may thus impose both women and their children to increased risks. Anecdotal evidence from doctors in Sweden indicates that clinical practice differs, some withdraw the drug, others continue.

In order to get reliable data on drug prescription, we asked the National Board of Health and Welfare to do a cross-match of the National Prescribed Drug Register and the National Medical Birth Register for 45 different drugs that, according to the European Network of Teratology Information Services, lacked sufficient scientific evidence for use during pregnancy and breastfeeding. A threshold of 500 prescriptions/year for entire Sweden was set. 12 drugs met the criteria, and among them, prednisolone was identified. Prednisolone was chosen as it was considered very important by the patient representatives and since collaborators within the ConcePTION project are performing in vitro studies on the drug. This study is performed as a part of the ConcePTION project, together with several other lactation studies.^5 6^

When treating breastfeeding women, not only is the mother exposed to potential risks of treatment but possibly also the breastfeeding infant. Hence, it is important to fully understand any risks that the breastfeeding infant is exposed to due to maternal medical treatment. This is essential to be able to weigh the benefits of treatment against its risks, and for clinicians and women to make well-informed decisions concerning treatment and breastfeeding. Previous studies assessing both secretion of prednisolone in breast milk and in plasma are few and typically include small numbers of women/infants. Until today, only a handful of publications have studied how maternal prednisolone treatment affects breastfeeding and the breastfeeding infant.^7^ To better understand any risks that the breastfeeding child is exposed to, it is important not only to study the concentration of prednisolone in breast milk but also the levels of prednisolone in the plasma of the breastfeeding child, and potential health effects in the infant. One possible side effect of prednisolone is decreased endogenous cortisol levels,^8^ which has not been studied in infants exposed to maternal prednisolone through breast milk. There is a need to systematically collect data to enable additional, larger studies on the transfer of prednisolone into breast milk and to the breastfeeding infant.

### What is already known?

We have identified clinical publications, covering studies, which have measured secretion of prednisolone in breast milk and health-related aspects of the breastfeeding infants. An early study of seven women^9^ indicated a range from 0.07% to 0.23% of the dose is transferred into human breast milk, concluding that the amount received by the infant would be extremely small. In two patients tested at 14 weeks postpartum, prednisolone was 22-fold and 14-fold higher in maternal plasma as compared with breast milk, when analysed 3 hours after dose intake. Prednisolone was undetectable in breast milk, 6 hours after dose intake.^10^ One report of 6 women given 10–80 mg of prednisolone daily found that minimal amounts of prednisolone were transferred to breast milk at doses under 20 mg. The study estimated that at the highest dose, less than 0.1% the maternal dose would be ingested by the nursing infant.^11^

In another study, prednisolone transfer to breast milk was studied in 3 women given 50 mg of prednisolone sodium phosphate intravenously.^12^ 30 minutes after injection, milk prednisolone concentrations varied from about 200 to 400 µg/L and dropped with a half-life slightly faster than the 2.5 hour half-life in serum. An average of 0.025% of the prednisolone was recovered in milk, suggesting that transfer to breast milk does not have a clinically significant risk to nursing infants.^12^ In a series of 124 transplant recipients who nursed while taking low doses of prednisone, none of the infants had health issues.^13^ From these studies, relative infant dose (RID) could be calculated to be as low as 0.1% but also as high as 7.3% if assuming a worst-case scenario.^10 14 15^

Prednisolone is a small molecule (340 Da) that is neutral in pH, has low solubility in water and high solubility in fat. Small molecules usually pass easily into breast milk, which would indicate a higher transfer. With high fat solubility, it is expected that higher concentrations are found in hind milk. The dose-dependent protein binding in plasma is likely the most important predictor of transfer into breast milk. By using a full portion of breast milk, the difference between hind milk and fore milk is considered to be less important.

According to Food and Drug Administration (FDA) recommendations for clinical lactation standards, there are several study designs that may be used related to the burden of data collection on the mother while still obtaining adequate data.^16^ Access to the study population, half-life of the drug, stability of the drug during collection procedures and possibility to provide pharmacokinetic data are other criteria for selecting an appropriate study design. Different designs (milk-only, mothers’ milk and plasma, mother–infant pair) are possible and permitted. Real world data on the transfer of medicine to an infant in association with breastfeeding requires plasma sampling from the infant. We demonstrate how the study is designed, the methodology, sampling procedures, informed consent procedures, data handling and ethical requirements are managed. Following the FDA recommendations, lactation studies should preferentially not be initiated earlier than 6 weeks after delivery, to allow the mother to go back to a normal physiology and to not interfere with the establishment of breastfeeding and mother–child bonding.

The project is intended to demonstrate how biobanking and sampling of breast milk and blood plasma can be done in order for the obtained analysis results to meet regulatory requirements from the European Medicines Agency (EMA) and FDA, respectively.^16 17^ This can be of great benefit for all other situations where there is currently not sufficient evidence regarding the transfer of a medicine to the child during breastfeeding, something that applies to almost all medicines. By establishing a sample collection with breast milk and associated samples of blood plasma, following approved information and consent procedures for future research, there is also an opportunity to investigate in the future how, for example, genetic factors can affect drug transfer. Other yet not planned studies can be performed without additional sampling. Data collection is still ongoing.

## Methods and analysis

### Study design

A low-intervention clinical trial with biobanking of breast milk and plasma. To assess the potential transfer of prednisolone to the infants of breastfeeding mothers, we set up a mother–infant pair lactation study with sampling of breast milk, mothers’ plasma and infants’ plasma. The study was deemed a low-intervention clinical trial as it evaluates the effects of pharmacological treatment and includes plasma samples of newborns which are not taken on a routine basis at the desired period of 6–8 weeks postpartum.

### Objectives

The primary objective is to determine the concentration of prednisolone in the plasma of breastfed infants of lactating women treated with prednisolone. Secondary objectives are to determine the concentration of prednisolone in the breast milk and maternal plasma and the milk-to-plasma ratio in the mothers and to calculate the daily infant dose (DID) and relative infant dose (RID). A third objective is to evaluate the cortisol levels in the infants. Since prednisolone is very low in plasma and breast milk after 4–6 hours, we aimed to analyse concentrations at peak levels.

### Patient selection

#### Inclusion criteria

Adult (≥18 years of age) breastfeeding mothers treated with prednisolone (50 mg/day or less) for clinical reasons, and their newborn infants. Participation in the study does not in any way impact or interfere with the patients’ treatment as prescribed by their physician.

#### Exclusion criteria

Mother not able to understand Swedish or English, multiple births (twins, triplets or more), preterm birth (<37 weeks gestation). Infants treated with prednisolone will not be included.

#### Intervention

Clinical practice is followed in all procedures, and no intervention is given.

The indication of prednisolone is to reduce inflammation in a variety of immune-related diseases, inflammatory diseases and allergic diseases. The dose of prednisolone needs to be stable for at least a week before the sampling takes place.

### Sample size calculation

No formal sample size calculations have been performed as there are no statistical hypotheses to be tested. The decision on the sample size was based on considerations including pharmacokinetic and pharmacodynamic variability for the drug, and the variability in lactation physiology including intersubject and intrasubject variability for mother and the breastfed child.^11 12 18^ The considerations were made in accordance with the FDA lactation studies guidance from 2019 and 2005 where six–eight subjects were considered to suffice in milk-only studies. Furthermore, the choice to analyse peak concentrations and to administer all doses together with food was considered to reduce effects of intrasubject and intersubject variability. A sample size of 25 pairs were considered to be sufficient. Given that approximations had to be used, the sample size was increased to 30.

### Recruitment

General information about the project is provided through a network of healthcare professionals, via *Janusmed* at Region Stockholm^15^ and through information in social media. In collaboration with *the Rheumatism Association* who distributed information about the study to their members and specialist antenatal care units, patients treated with prednisolone, regardless of diagnosis (eg, rheumatoid arthritis, systemic lupus erythematosus, myositis, psoriatic arthritis, inflammatory bowel disease) are informed about the study with written information as well as a video describing the project. A link is provided to the project webpage for more information.

Women willing to participate in the study are provided with detailed study information, including an opportunity to ask questions about what this entails and provided with a written informed consent form, which they will sign and also ask their partners (the other caregiver) to sign. The partner’s signature is a requirement for taking blood samples of the child. A copy of the subject information as well as the informed consent form is provided to the woman. In the research subject information, the mother is informed that when providing a blood sample, you usually feel a slight sting. You can get a bruise, and there is always a small risk of nerve damage, which can be expressed as pain or numbness. The research subjects (women and infants) are not expected to benefit directly from participating in the study. However, the study results will be able to contribute information that in the future can help breastfeeding women to make an informed decision about using prednisolone in connection with breastfeeding. Such information will also help healthcare professionals to provide evidence-based advice to breastfeeding women about the use of prednisolone.

Each subject who participates in the trial is identified by a subject number on a subject identification list.

Discontinuation for individual subjects is based on withdrawal of consent. Withdrawal is made by notifying the local clinical staff or via the study website. Enrolment will be stopped after 30 women have completed their participation in the study. The principal investigator at each site will ensure that the subject is given full and adequate oral and written information about the trial, its purpose, any risks and benefits as well as inclusion and exclusion criteria. Subjects are informed that they are free to discontinue their participation in the trial at any time without having to provide a reason.

### Setting

Uppsala University is the sponsor of the study, with funding via the IMI-ConcePTION project (Innovative Medicines Initiative 2 Joint Undertaking, Grant agreement No. 821520). Taking plasma samples from infants at 6–8 weeks postpartum requires close collaboration with experienced and dedicated clinical centres. Clinical trial sites with approved local trial investigators are: Astrid Lindgren Children’s Hospital in Stockholm, Södra Älvsborgs Hospital in Borås and Umeå University Hospital. The trial is designed in compliance with the EU regulation on low-intervention clinical trials on medicinal products for human use (536/2014), the Declaration of Helsinki, ICH-GCP standards applicable to the trial procedures, and current national regulations governing this clinical trial.

### Sampling procedures

**Figure 1:**
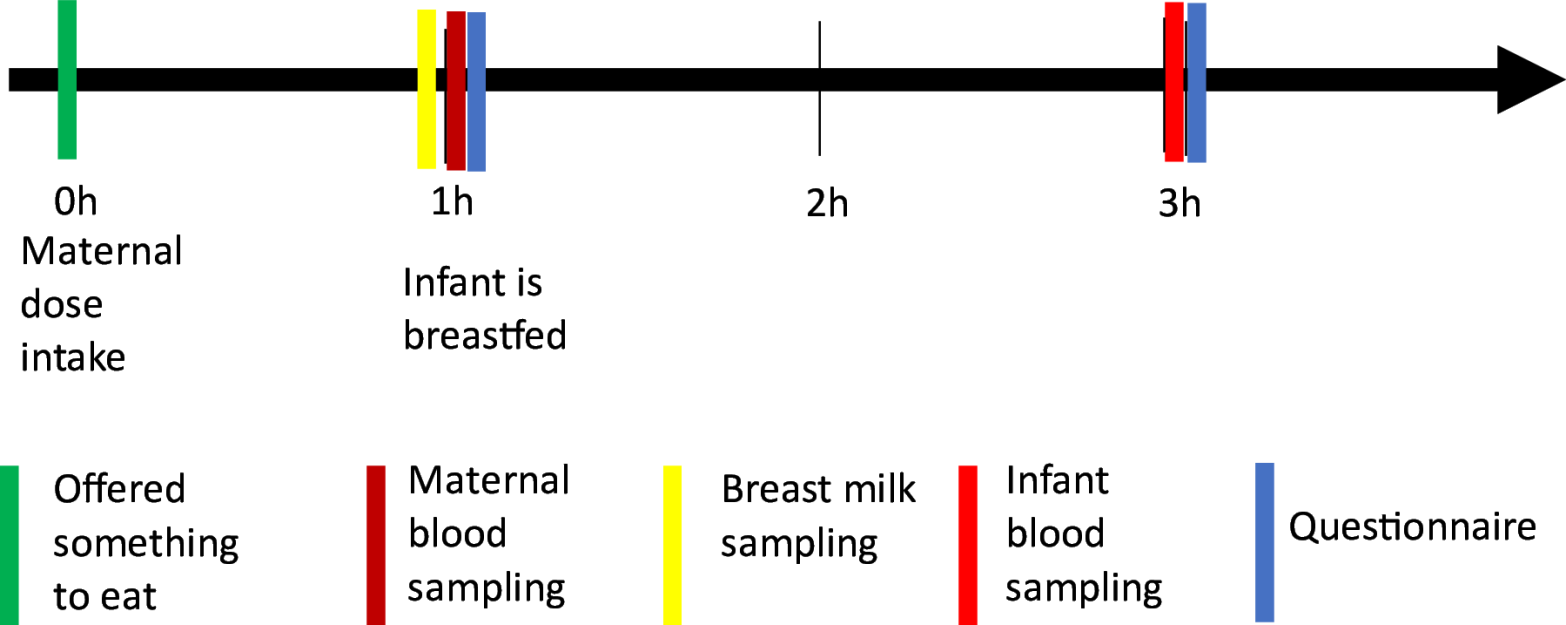
Overview of sampling procedures. The study visit starts with offering the mother something to eat, before prednisolone intake (0 hours). This is followed by a maternal blood sample (1 hour), a breast milk sample (1 hour), feeding the infant breast milk (1 hour) and responding to online questionnaires (1 and 3 hours). Blood sampling from the child is done 2 hours after breastfeeding (3 hours after maternal dose). The whole procedure takes place in the morning, before lunch.

Women accepting participation are scheduled for a study visit. Sampling takes place after the development of mature milk, at least 6 weeks postpartum. The sampling takes place after achieving steady state; after having been on prednisolone without any dosage changes for a minimum of 7 days. All sampling takes place the same day at any of the clinical sites. The mother brings her own prednisolone medication to the clinic. A timeline of the study visit is shown in figure 1.

Blood sampling is performed according to local routines and manufacturer instructions. At sampling from the mother, two LiHep tubes (7 mL each) are filled. If the participant already has a venous catheter she prefers to use, an additional slush tube (7 mL) with blood is drawn. In total, 1×2×7 mL (14 mL) is drawn from each participating mother (21 mL if an existing catheter is used). From the infant, 0.8 mL of blood is sampled. This can be compared with the 450 mL of blood during a normal blood donation.

Blood sampling in children is associated with pain. The clinically scientifically supported routine that the project follows is to take a venous blood sample on the back of the hand. In connection with this, *emla* (lidocaine/prilocaine) and/or sugar solution is given.^19^ This blood sampling is done by experienced healthcare personnel.

Data associated with the sampling procedure and which other drugs the woman is taking are documented. Sampling details (every sampling) and health-related details concerning the mother and her infant (every sampling) are collected using an online questionnaire. If a blood status is collected from the infant as clinical routine, permission will be requested to obtain this information from the medical journal.

Breast milk is sampled at one timepoint: 1 hour after maternal prednisolone intake. The breast milk sampling is carried out by the mother using an electric pump she receives from the project. The electric pump is of the same brand and model for all participants. Full milk expression from at least one of the breasts is mixed well, and a 10–20 mL sample is taken. After expression, the mother feeds the infant the remaining milk that was expressed (and the milk from the other breast if only one is expressed by the pump for sampling).

The blood and breast milk samples are transported to the local lab after sampling. The blood sample will be centrifuged (2000 g, 10 min) and aliquoted. The plasma aliquots are frozen and then shipped on dry ice to Uppsala Biobank.

The collected milk sample is aliquoted at the local lab and put on dry ice for shipping. All aliquots are stored at −80°C in Uppsala Biobank prior to analyses being performed.

The quantification of prednisolone concentrations in human breast milk and maternal and infant plasma will be performed using liquid chromatography–mass spectrometry (LC-MS/MS, Waters Corp, Milford, Massachusetts, USA).

Sample analysis will be carried out by taking 40 µL of sample (breast milk or plasma) and precipitating proteins by adding three volumes of acetonitrile containing an internal standard in a 96-well plate. After centrifugation, 60 µL of the supernatant will be transferred to a new sample plate and diluted with 110 µL of water containing an internal standard. The sample plate will then be transferred to the ultra-high performance liquid chromatography (UHPLC)-MS/MS system for analysis.

A Waters Xevo TQ-S micro (triple quadrupole mass spectrometer), coupled with a Waters Acquity UHPLC used for chromatographic separation on an Acquity BEH C18 column (inner diameter 2.1×50 mm, particle size 1.7 µm, pore size 130 Å), will be used. The mobile phases will consist of 0.1% (v/v) formic acid in water (mobile phase A) and acetonitrile (mobile phase B). Chromatographic separation will be completed in 3 min per sample at a flow rate of 0.7 mL/min.

A TQ-S micro equipped with an electrospray ionisation source will be used for detection in positive ion mode. Quantification will be based on multiple reaction monitoring of the transitions m/z 361.16>146.996 for prednisolone and m/z 369.33>124.92 for prednisolone-d8. A linear regression model with 1 /x² weighting will be used to construct a calibration curve with linear ranges between 1.80 and 541 ng/mL in both milk and plasma. Lowest level of quantification is estimated to 10 nmol/L.

Data acquisition will be performed using MassLynx software (V.4.2, Waters Corp). Compound peaks will be integrated and quantified with TargetLynx (V.4.1, Waters Corp) based on the peak area ratio of the target compound to the internal standard, using linear regression.

The validated methods were able to quantify prednisolone with precision and accuracy within regulatory guidelines (CV <15%, bias ±15%). Deuterated internal standards were selected for all analytes to ensure accurate quantification. Short-term stability at room temperature was confirmed for up to 72 hours, and for 7 days at 4°C. Long-term stability at −20°C and −80°C was confirmed for up to 365 days. Appropriate quality control (QC) samples were established and applied for accuracy and precision assessments. These QC samples will be included in all future analyses to ensure compliance with validated standards. Selectivity and specificity were evaluated in all matrices (human breast milk and plasma), and the matrix effect was assessed, all within acceptance criteria according to regulatory guidelines. No interference from endogenous compounds or metabolites was observed. In all analytical runs of samples from lactation studies, blank samples were included to ensure that no carry-over effects occurred.

### Calculation

Dosage of infant as DID in mg/kg/day will be estimated by using 200 mL/kg/day as a standard for daily milk intake. This is in accordance with FDA guidelines for lactation studies^16^: ‘While a 150 mL/kg/day estimated milk intake is a reasonable assumption to estimate daily infant dosage, greater volumes do occur in early infancy and often correlate to the time of most reported infant adverse drug events. Additional consideration should be given to estimates of infant risk based on a 200 mL/kg/day milk intake in early infancy’.


$$DID\left(mg/kg/d\right)=\frac{milk\;concentration\left(mg/L\right)\times milk\;volume\left(L\right)}{infant\;weight\left(kg\right)}$$

$$RID\left(\%\right)\frac{Daily\;Infant\;Dose\left(mg/kg/d\right)}{daily\;maternal\;dose\left(mg/d\right)/maternal\;weight\left(kg\right)}\;\times 100$$

RID will be calculated according to the equation above. It is estimated that a RID <10% is considered safe for the infant.^20^ However, several other factors affect the safety of drug use during lactation such as the half-life of the drug, active metabolites, toxicity and effects of the drug and the infant's capacity to eliminate the drug.^21^ In this case, prednisolone may possibly affect the infant’s cortisol levels also after the drug has been eliminated. Calculations of DID and RID will rather reflect the highest possible dose, as sampling is made close to peak plasma levels.

The maternal prednisolone milk to plasma ratio will be calculated by dividing drug concentration in the mother’s milk with the drug concentration of the mother’s plasma.

Prednisolone concentration in infant blood will be reported, and infant/mother plasma ratio will be calculated.^21^ Cortisol concentration in infant blood will also be reported.

### Ethics and dissemination

The clinical trial was approved by the Swedish Medical Product Agency (Dnr. 5.1.1-2023-104170) (online supplemental file 1). The clinical trial protocol is available via the Clinical Trial Information System at the EMA (No. 2023-508913-18-00). It is also registered and publicly accessible at the EU PAS Register (EUPAS 1000000059, CTIS 2023-508913-18-00). The results will be published in peer-reviewed scientific journals and be presented at scientific meetings. A popular science description will be published online at the website of The Swedish Rheumatism Association.

### Data protection

In the study information provided, participants are fully informed about how their trial data will be used. The content of the informed consent form complies with relevant integrity and data protection legislation, including the Regulation (EU) 2016/679 (General Data Protection Regulation (GDPR)). The subject information and the informed consent form explain how trial data are stored to maintain confidentiality in accordance with national data legislation and GDPR. All information processed by the sponsor is pseudonymised and coded. The code key is stored at the clinical trial sites while the study is ongoing and then securely transferred to Uppsala University in an encrypted version.

Sampling details and health-related details are collected using a paper questionnaire and then registered in the programme REDCap (Research Electronic Data Capture). Informed consent forms are sent by registered mail to Uppsala University and stored in a locked cabinet only accessible by authorised personnel. Data are uploaded in an encrypted format, which then is hosted at a locked and secure server of Uppsala University (Dataportal ALLVIS) and Region Uppsala in accordance with an established process approved by the Swedish Ethical Review Authority.

### Patient and public involvement

The Swedish Rheumatism Association was involved in the preliminary discussions about the study design. The Association deemed information about prednisolone during breastfeeding as a priority, which influenced the choice of prednisolone for this demonstration study. Information about the study was published on the website to inform members and to facilitate recruitment. Results from the study will be published in a popular science version on the website.

### Preliminary findings

Up to date (1 April 2025), eight women and infants have been recruited and sampled in Stockholm, three women and infants in Borås and one in Umeå. These samples have been transferred to Uppsala Biobank and delivered to Uppsala University Drug Optimization and Pharmaceutical Profiling Platform (UDOPP) for analysis. Recruitment is ongoing at all sites. Evaluation of clinical experiences related to recruitment and sampling will be systematically collected at the completion of the study, which is estimated at the end of 2025. Experiences so far indicate that the greatest challenge for a study like this is recruitment of a sufficient number of participants.

## Supplementary material

10.1136/bmjopen-2024-097898 online supplemental file 1
