# Supplementary material for: Determination of prednisolone concentration in human breast milk and plasma of breastfed infants: study protocol of a Swedish multicentre low-intervention clinical trial
Source: BMJ Open. 2025 May 22;15(5):e097898. doi: 10.1136/bmjopen-2024-097898 (PMC12097052; doi:10.1136/bmjopen-2024-097898)
Supplement: online supplemental file 1 [file bmjopen-15-5-s001.pdf]

CLINICAL TRIAL PROTOCOL

Transfer of prednisolone into human breast milk and plasma of breastfeeding children - A low intervention cohort study with biobanking of breast milk and plasma

EU Trial number:	2023-508913-18-00
Version number:	5.1
Date:	2023-12-08
Sponsor:	Uppsala University<br>Centre for Research Ethics & Bioethics,<br>Department of Public Health and Caring Sciences,<br>Uppsala University, Box 564,<br>SE-751 22 Uppsala, Sweden

Table Of Contents

Description of changes in the trial protocol 5
Signature page 6
Contact information 7
List of used acronyms and abbreviations 8
1. Swedish synopsis 10
2. Background and rationale 12
3. Benefit-risk evaluation 13
4. Trial objectives 14
4.1. Primary objective 14
4.2. Secondary objective(s) 14
4.3. Primary endpoint 14
4.4. Secondary endpoint 14
5. Trial design and procedures 14
5.1. Overall trial design 14
5.2. Procedures and flow chart 15
5.3. Biological sampling procedures 15
5.3.1. Handling, storage, and destruction of biological samples 16
5.3.2. Total volume of blood per subject 16
5.3.3. Biobank 16
5.4. End of Trial 17
6. Subject selection 17
6.1. Inclusion criteria 17
6.2. Exclusion criteria 17
6.3. Selection of subjects 17
6.4. Withdrawal criteria 18
7. Trial treatments 18
7.1. Description of investigational medicinal product(s) 18
7.2. Dose and administration 18
7.3. Drug accountability and treatment compliance 18
7.4. Randomization and blinding 19
7.5. Concomitant use of other medicinal products and treatments 19

7.6. Treatment after trial end 19
8. Methods for measurement of endpoints for clinical efficacy and safety 19
8.1. Methods for measurement of endpoints for clinical efficacy 19
8.2. Methods for measurement of endpoints for clinical safety....19
8.2.1. Specification of pharmacological safety parameters 19
9. Handling of Adverse Events 20
9.1. Definitions....20
9.1.1. Adverse Event (AE) 20
9.1.2. Adverse Reaction (AR) 20
9.1.3. Serious Adverse Event (SAE) 20
9.1.4. Suspected Unexpected Serious Adverse Reaction (SUSAR)....21
9.2. Assessment of Adverse Events (AE) 21
9.2.1. Assessment of seriousness 21
9.3. Reporting and registration of Adverse Events....21
9.3.1. Reporting of Serious Adverse Events (SAE) 21
9.3.2. Reporting of Suspected Unexpected Serious Adverse Reactions (SUSAR)....21
9.4. Follow-up of Adverse Events 21
9.5. Independent Data Monitoring Committee....21
9.6. Annual Safety Report (ASR)....22
9.7. Procedures in case of emergencies, overdose or pregnancy....22
10. Statistics....22
10.1. Analysis population....22
10.2. Statistical analyses 22
10.2.1. Statistical methods....22
10.2.2. Drop-outs....23
10.3. Sample size calculations 23
10.4. Interim analysis....23
11. Quality Control and Quality Assurance 23
11.1. Quality Assurance and Sponsor oversight 23
11.2. Monitoring....23
11.3. Source data 24
11.4. Deviations, serious breaches and other reporting obligations 25

11.5. Audits and inspections....25
12. Ethics....25
12.1. Compliance to the protocol, ICH-GCP and regulations....25
12.2. Ethical review of the trial....25
12.3. Procedure for obtaining informed consent....26
12.4. Data protection....26
12.5. Insurances....26
13. Substantial changes to the trial....26
14. Collection, handling, and archiving of data....27
14.1. Case Report Form....27
15. Notification of trial completion, reporting, and publication....27
16. References....27
17. Attachments....28

Description of changes in the trial protocol

Protocol version	**Summary of changes**<br>*Describe all changes since the first final protocol.*
5.0	Komplettering i avsnitten 5.4, 9 och 11 visavi version 4.0
5.0	Bifogade attachments under punkt 16

Signature page

Sponsor

I am responsible for ensuring that this protocol includes all essential information to be able to conduct this trial. I will submit the protocol and all other important trial-related information to the responsible investigator(s) so that they can conduct the trial correctly. I am aware that it is my responsibility to hold the staff members who work with this trial informed and trained.

Sponsor’s signature Date

Mats Hansson
Senior Professor

Principal Investigator

I have read this protocol and agree that it includes all essential information to be able to conduct the trial. By signing my name below, I agree to conduct the trial in compliance with this clinical trial protocol, the EU Regulation on clinical trials of medicinal products for human use (EU 536/2014), the Declaration of Helsinki, ICH-GCP (Good Clinical Practice) guidelines and the current national regulations governing the conduct of this clinical trial.

I will submit this protocol and all other important trial-related information to the staff members and investigators who participate in this trial, so that they can conduct the trial correctly. I am aware of my responsibility to continuously keep the staff members and investigators who work with this trial informed and trained.

I am aware that quality control of this trial will be performed in the form of monitoring and eventual audit and inspection.

Principal Investigator’s signature Date

Jenny Svedenkrans,
Senior Consultant of Neonatology, Pediatrician,

Contact information

**Responsibility in the clinical trial**	
Sponsor	Uppsala University<br>Mats Hansson, Senior Professor<br>Address: Centre for Research Ethics & Bioethics, Department of Public Health and Caring Sciences, Uppsala University, Box 564, SE-751 22 Uppsala, Sweden<br>Email: [redacted]<br>Phone: [redacted]
Principal Investigators	Jenny Svedenkrans, Senior Consultant of Neonatology, Pediatrician,<br>Karolinska Universitetssjukhuset, Stockholm<br>Phone: [redacted]<br>Erika Timby, Senior Consultant, Maternity Care,<br>Kompetenscentrum för Mödra- & Barnhälsovård Region Västerbotten,<br>Phone: [redacted]<br>Karin Backlund, Resident physician, Pediatric care unit, Södra Älvsborg Hospital<br>Phone: [redacted]
Clinical laboratory	Clinical chemistry and pharmacology Uppsala University Hospital, SE-751 85 Uppsala
Biobank	Uppsala Biobank, Dag Hammarskjölds väg 38, 5tr., UCR, Uppsala Science Park, Hubben<br>751 85 Uppsala

List of used acronyms and abbreviations

Abbreviation	Term/Explanation
ADID	Average Daily Infant Dose
Adverse Event (AE)	Any untoward medical occurrence in a subject to whom a medicinal product is administered and which does not necessarily have a causal relationship with this treatment.
AR	Adverse Reaction = any unfavorable and unexpected reaction to an investigational medicinal product, regardless of dose
ASR	Annual Safety Report = the annual safety report for reporting to authorities. In Sweden this is the Swedish Medical Products Agency via CTIS.
BLQ	Below the limit of quantification
CA	Competent Authorities
CRF	Case Report Form
CTIS	Clinical Trials Information System = Centralized EU database/portal for application and communication with authorities concerning clinical trials. In Sweden this includes the Swedish Medical Products Agency and the Swedish Ethical Review Authority.
ENTIS	the European Network of Teratology Information Services
ICH-GCP	International Council for Harmonization Good Clinical Practice
LLOQ	lower limit of quantification
NMBR	the National Medical Birth Register
NPDR	the National Prescribed Drug Register
REDCAP	Research Electronic Data Capture
RID	Relative infant dose

Serious Adverse Event (SAE)	Any untoward medical occurrence that at any dose requires inpatient hospitalisation or prolongation of existing hospitalisation, results in persistent or significant disability or incapacity, results in a congenital anomaly or birth defect, is life-threatening, or results in death
SD	Standard deviation
SUSAR	Suspected Unexpected Serious Adverse Reaction. This is an event that is likely related to the investigational medicinal product but with unexpected occurrence. An adverse reaction is unexpected if its nature or seriousness is not consistent with the information on the product in the RSI.

1. Swedish synopsis

EU prövningsnummer:	2023508913-18-00
Titel:	Överföring av prednisolon till bröstmjölk och blodplasma från ammande barn - En låginterventionsstudie med biobankning av bröstmjölk och blodplasma
Kort bakgrund/ Rational/Syfte:	För många läkemedel saknas idag tillräckliga underlag för hur läkemedlet kan påverka barnet som ammas. Det har i många fall inneburit att man av försiktighetsskäl sätter ut läkemedlet eller rekommenderar mamman att inte amma. Samtidigt har mamman bäst nytta av det förskrivna läkemedlet och man vet att amning är bra både för det nyfödda barnet och för mamman. Syftet med studien är att undersöka om och hur mycket av prednisolon som går över via bröstmjölken till barnet från mammor som har fått läkemedlet förskrivet.
Primärt syfte:	Det primära syftet är att bestämma koncentrationen av prednisolon i plasman hos ammade spädbarn till ammande kvinnor som behandlats med prednisolon för att minska inflammation.
Sekundärt syfte:	Sekundärt mål är att bestämma koncentrationen av prednisolon/prednison i bröstmjölken och moderns plasma och mjölk-till-plasmaförhållandet hos mödrarna och att beräkna den genomsnittliga dagliga spädbarnsdosen (ADID) och relativ spädbarnsdos (RID).
Primärt utfallsmått:	Det primära utfallsmåttet är koncentrationen av prednisolon i det ammande barnets plasma 2 timmar efter att ett spädbarn matats med bröstmjölk, med utfodringen 1 timme efter moderns dosintag.
Sekundärt utfallsmått:	De sekundära effektmåtten är koncentrationen av prednisolon och prednison i bröstmjölk 1 timme efter moderns dosintag och moderns plasmakoncentration av prednisolon och prednison 1 timme efter prednisolonintag.
Prövningsdesign:	Studien har en låg interventionell design i den meningen att bröstmjölk och blod samlas in enbart för

	att studera utsöndringen av prednisolon i bröstmjölken och överförning till det ammade barnet.
Prövningspopulation:	Ammande kvinnor över 18 år som behandlas med prednisolon och deras nyfödda barn (6-8 veckor efter födseln)
Antal försökspersoner:	30
Inklusionskriterier:	Ammande kvinnor som behandlas med prednisolon med doser upp till 50 mg/dag för alla tillstånd. Endast kvinnor från 18 år och äldre kommer att tillfrågas om att delta. Ammande barn till kvinnor som har samtyckt till att delta i studien. Vårdnadshavare till barnet tillfrågas och informerat samtycke inhämtas också från fadern
Exklusionskriterier:	Kvinnor som inte kan läsa och kommunicera på svenska eller engelska. Kvinnor med spädbarn födda före 37 veckors graviditet (för tidig). Kvinnor med flerbörd.
Intervention:	Ingen behandling förekommer i studien, se ovan.
Prövningsläkemedel, dosering, administrering:	Prednisolon med doser upp till 50mg/dag i enlighet med förskrivning av läkare.
Etiska överväganden, nytta/risk:	Risk-nytta-balansen avseende prednisolon påverkas inte av att delta i studien. Provtagningen görs enligt etablerade kliniska rutiner. Man känner ett lätt stick när man tar ett blodprov. Man kan få ett blåmärke och det finns alltid en liten risk att påverka en nerv, vilket kan uttryckas som smärta eller domningar. Vid blodprov tas totalt 14 ml blod vid ett tillfälle. Detta kan jämföras med de 450 ml blod som tas under en normal blodgivning. Försökspersonerna förväntas inte ha någon direkt nytta av att delta i studien. Studieresultaten kommer dock att kunna bidra med information som i framtiden kan hjälpa ammande kvinnor att fatta ett välgrundat beslut om användning prednisolon i samband med amning. Sådan information kommer också att hjälpa vårdpersonal att ge evidensbaserade råd till ammande kvinnor om användningen av prednisolon.
Prövningsperiod:	Q1 2024-Q4 2024, Publicering inom ett år efter studiens avslut.

2. Background and rationale

More than 5 million women get pregnant in the EU every year and a majority take at least one medication during pregnancy. As few as 5% of available medications have been adequately monitored, tested and labelled with safety information for use in pregnant and breastfeeding women. The field, while inherently difficult to study, has suffered from a lack of systematically gathered insights that could lead to more effective data generation methodologies. Fragmentation and misinformation results in confusing and contradictory communication and perception of risks by both health professionals and women and their families.

In clinical settings doctors often take a precautionary approach and discontinue the prescription of a given drug during pregnancy and breast feeding, or recommend to stop breast feeding. There is a cost of this approach, both for the woman and for her child. The woman is in need of the drug, which may not be replaceable by another drug, and one knows that breast feeding is good, both for the mother and for the child. The lack of evidence regarding transfer of drug to the breastfeeding infant may thus impose both women and their children to increased risks.

Anecdotal evidence from doctors in Sweden indicate that clinical practice differs, some withdraw the drug, others continue. In order to get reliable data on prescription, we asked The National Board of Health and Welfare to do a cross match of the National Prescribed Drug Register (NPDR) and the National Medical Birth Register (NMBR) for 45 different drugs that according to the European Network of Teratology Information Services (ENTIS) lacked sufficient scientific evidence for use during pregnancy and breastfeeding. A threshold of 500 prescriptions/year for entire Sweden was set. Among the 12 drugs that so were identified was *prednisolone*. The uptake of the drug is spread all over Sweden so women taking *prednisolone* live in different parts throughout Sweden.

When treating lactating women who continue to breastfeed, not only the mother is exposed to the risks of treatment but potentially also the breastfeeding child. Hence, it is important to fully understand any risks that the breastfeeding child is exposed to due to maternal medical treatment. This is essential in order to be able to weigh the benefits of treatment against its risks, and for clinicians and women to make well-informed decisions concerning treatment and breastfeeding.

Until today, only a handful of publications have studied how maternal prednisolone treatment affects breastfeeding and the breastfeeding child. To better understand any risks that the breastfeeding child is exposed to, it is important to not only study the concentration of prednisolone in breast milk but also the levels of prednisolone in the plasma of the breastfeeding child. The previous studies that have studied excretion of prednisolone in breast milk and in blood mainly consist of case studies involving few lactating women and generally a smaller number of infants. There is a need for additional studies involving larger numbers of study participants, with systematically collected data.

3. Benefit-risk evaluation

Prednisolone is prescribed by the treating physician prior to subject enrollment in the trial. There is no change regarding the prescription or dosing of prednisolone after enrollment and the risk-benefit balance regarding prednisolone is thus not affected by participating in the trial.

Summary of benefits and risks in accordance with the Assessment Report from EMA (CHMP) 13 October 2016, EM/867221/2016:

The benefits of prednisolone when used to lower the activity of the immune system and prevent the release of substances in the body that cause inflammation and swelling have been demonstrated.

The most common side-effects observed in association with prednisolone use include fluid retention, dizziness, headache, muscle pain or weakness, bloating, weight gain, heartburn, insomnia, hunger, nausea or thrush. Prednisolone can weaken the immune system, making it easier to get an infection. Steroids can also worsen a current infection, or reactivate a recent infection.

The sampling during the trial is done according to established clinical routines. As stated in the research subject information, one usually feels a slight sting when taking a blood sample. One can get a bruise and there is always a small risk of affecting a nerve, which can be expressed as pain or numbness. When taking a blood sample, a total of 14 ml of blood will be collected at one occasion. This can be compared to the 450 ml of blood taken during a normal blood donation.

The research subjects are not expected to benefit directly from participating in the study. However, the study results will be able to contribute information that in the future can help breastfeeding women make an informed decision about using prednisolone in connection with breastfeeding. Such information will also help healthcare professionals provide evidence-based advice to breastfeeding women about the use of prednisolone.

An alternative to obtain data regarding the transfer of drugs during breastfeeding is to only collect breast milk. However, it is only when one also takes a blood sample from the breastfed child that one will get reliable data on how much of the medicine is actually transferred to the child. This is also the "golden standard" that applies to lactation studies internationally. Blood sampling in children is associated with a pain problem. The clinical scientifically supported routine that the project follows is to take a venous blood sample on the back of the hand. In connection with this, emla and/or sugar solution is given. This blood sampling is done by experienced and specially trained healthcare personnel. Only data associated with the sampling procedure and which other drugs the woman is on are documented. Privacy risks are further minimized by pseudonymisation.

4. Trial objectives

4.1. Primary objective

The primary objective is to determine the concentration of prednisolone in the plasma of breast-fed infants of lactating women treated with prednisolone to reduce inflammation in a variety of conditions.

4.2. Secondary objective(s)

Secondary objectives are to:

1. Determine concentration of prednisolone/prednisone in the breast milk and maternal plasma and the milk-to-plasma ratio in the mothers and to calculate the average daily infant dose (ADID) and relative infant dose (RID).
2. Evaluate the cortisol levels in the infants.

4.3. Primary endpoint

The primary endpoint is the concentration of prednisolone in the breastfeeding child's plasma 2h after feeding an infant breast milk, with the feeding taking place at 1h following maternal dose intake.

4.4. Secondary endpoint

The secondary endpoints are:

1. The concentration of prednisolone and prednisone in breast milk at 1h after maternal dose intake and the maternal plasma concentration of prednisolone and prednisone at 1h after prednisolone intake.
2. The concentration of cortisol in the infant blood.

5. Trial design and procedures

5.1. Overall trial design

Low intervention trial with collection and biobanking of breast milk and plasma.

Breast milk and blood will be collected merely to study excretion of prednisolone into breastmilk and transferal to her child. Participation in the trial will not decide or in any other way interfere with patients' treatment as prescribed by their physician. Only patients that already have been assigned treatment with prednisolone by their physician and made the choice to breastfeed, will be approached and asked for participation. All procedures for blood collection will follow established routines at the clinical sites.

The sampling will take place ≈ 6-8 weeks after birth at the designated clinic. At this time point it is estimated that there is nothing left from medication taken during pregnancy.

Women willing to participate in the study will be provided with detailed information about what this entails and provided with a written informed consent form, that they will sign and also ask their partners (the other care giver) to sign. They will bring the signed informed consent form with them to the sampling center.

Breast milk will be collected once during the visit, using an electric breast milk pump. Venous blood will be collected from the woman and from the infant. After centrifugation, plasma samples and the breast milk sample will be shipped to Uppsala Biobank where it will be frozen and stored. Samples will be analysed for pharmacokinetic properties using mass spectrometry at the platform UDOPP, Department of Pharmacy, Uppsala University.

5.2. Procedures and flow chart

Overview sampling procedure

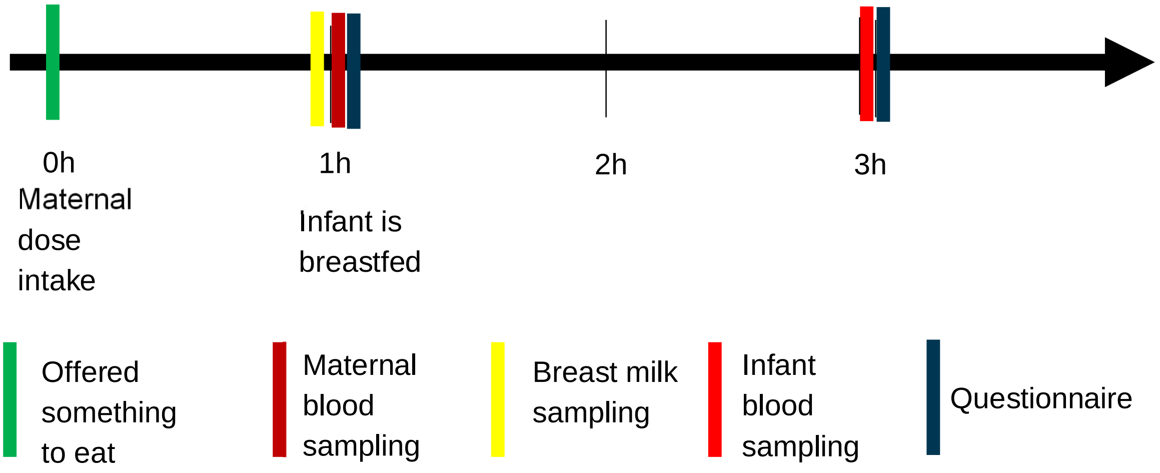

5.3. Biological sampling procedures

Women accepting participation are scheduled for breastmilk sampling. Sampling takes place after development of mature milk, at least six weeks postpartum. The sampling will take place after achieving steady state; after having been on prednisolone without any dosage changes for a minimum of 7 days. All sampling takes place the same day at any of the clinical sites. The mother brings her own prednisolone medication to the clinic.

Following FDA recommendations, lactation studies should preferentially not be initiated earlier than 6 weeks after delivery, to allow the mother to go back to a normal physiology and also to not interfere with establishment of breast-feeding and mother-child bonding. Participation starts with offering the mother something to eat, before prednisolone intake. Participants are expected to participate in the study until they have taken their dose of prednisolone (0h), provided a blood sample (1h), a breast milk sample (1h), followed by feeding their child breast milk, and responded to online-questionnaires (1 and 3h). Blood sampling from the child is done 2h after breastfeeding (3h after maternal dose).

The whole procedure is taking place in the morning, before lunch. As prednisolone is normally taken during or after having a meal, the mother should be offered something to eat before dose intake. Maternal blood sample is taken once; 1h after dose and at same time infant is fed.

Sampling is performed according to local routines and manufacturer instructions. At sampling, 2 LiHep tubes (à 7ml) are filled. If the participant already has a venous catheter she prefers to use, an additional slush tube (7 ml) with blood is drawn. In total, 1x2x7 ml (14 ml) is drawn from each adult participant (21 ml if an existing catheter is used).

Breast milk is sampled at one timepoint: 1h after maternal prednisolone intake. The breast milk sampling is carried out by the mother using an electric pump she receives from the project. The electric pump is of the same brand and model for all participants. Full milk expression from at least one of the breasts is mixed well and a 10- 20 ml sample is taken. After expression the mother feeds the infant the remaining milk that was expressed (and the milk from the other breast if only one is expressed by the pump for sampling).

A venous blood sample of the back of the hand is drawn from the child once, 3h after maternal dose intake. According to the scientific evidence and the clinical experiences this is the least painful type of blood drawn from infants (Shah & Ohlsson, Cochrane Library 2011). At sampling, 0.8 ml are drawn. 0.2 ml of the sample will be used for cortisol analysis. These sampling procedures follow established local clinical routines with adding of *emla* and/or sugar solution.

Sampling details (every sampling) and health-related details concerning herself and her child (every sampling) are collected using a questionnaire.

5.3.1. Handling, storage, and destruction of biological samples

The blood samples are transported to the local lab (in a temperature that is consistent with the stability conditions) for centrifugation and aliquoting. The aliquots are frozen at -20 C° and then shipped on wet ice to Uppsala Biobank.

The collected breast milk sample is immediately put in the freezer and stored at -20 C° before being shipped to Uppsala Biobank on wet ice for aliquoting. The aliquots are stored at -80 C° prior to analyses being performed.

5.3.2. Total volume of blood per subject

The total volume of blood taken from each subject during the trial is a maximum of 14 ml from the woman and 0.8 ml from the child.

5.3.3. Biobank

All samples taken in this trial are registered in at Uppsala Biobank (IVO no. 827) and handled according to the current biobank laws and regulations. The samples are coded/pseudonymized to protect the subject´s identity. All samples and the identification/code list are stored securely and separately to prevent access by unauthorized persons.

5.4. End of Trial

The trial will be completed after 30 women have completed their participation in the study.

The trial may be prematurely terminated if it this is necessary for safety reasons affecting the risk-benefit balance or if the recruitment of subjects cannot be met within reasonable time limits. The Swedish Medical Products Agency will be informed as soon as possible via CTIS, but no later than 15 days after trial termination.

Decisions on premature termination are taken by the sponsor.

6. Subject selection

Lactating women treated with prednisolone and their breastfed infants.

6.1. Inclusion criteria

To be included in the trial, subjects must meet all of the following criteria:

- Breastfeeding women treated with prednisolone with doses up to 50 mg/day for any condition. Only women of 18 years of age and older will be asked for participation.
- Breastfeeding children of women that have consented to participate in the study. The guardians of the child are asked for participation. Informed consent is also obtained from the father.

6.2. Exclusion criteria

Subjects must not be included in this trial if any of the following criteria are met:

- Women not able to read and communicate in Swedish or English.
- Women with infant born prior to 37 weeks gestation (premature).
- Women with multiple births.

6.3. Selection of subjects

Collection of samples will be made at three clinical sites, The Specialist maternity Clinic in Umeå, The maternity department at Södra Älvsborgs Hospital and the Specialist Clinic for Children and Young, at Liljeholmen/Astrid Lindgrens Barnsjukhus/Karolinska institutet.

General information about the project will be provided through a network of health care professionals via *Janusmed* at Region Stockholm and through information in social media. In collaboration with *the Rheumatism Association* and specialist antenatal care, patients treated with prednisolone, regardless of diagnosis (e.g., rheumatoid arthritis, SLE, myositis, psoriatric arthritis, inflammatory bowel disease) will be informed about the study with written information as well as a video describing the project. A link will be provided to the project webpage for more information.

Women willing to participate in the study will be provided with detailed information about what this entails and provided with a written informed consent form, that they will sign and also ask their partners (the other care giver) to sign. They will bring the signed informed consent form with them to the medical doctor at the sampling center.

6.4. Withdrawal criteria

Discontinuation for individual subjects is based on withdrawal of consent. Withdrawal is made by notifying the local clinical staff or via the study website. Samples will be destroyed upon withdrawal. Pharmakokinetic data will be stored and part of further analysis together with data from those remaining in the study.

Subjects can discontinue their participation in the trial at any time without any consequence to his/her continued treatment. The investigator/sponsor can at any time terminate the trial for a subject due to, e.g., unacceptable adverse events/adverse reactions or because the subject cannot follow procedures in the clinical trial protocol. If the subject discontinues the trial, follow-up of this subject will be performed according to the clinic's routine.

7. Trial treatments

No treatment or medical prescriptions will be part of the trial. The project has thus a low intervention trial design in the sense that breast milk and blood will be collected merely to study excretion of prednisolone into breastmilk and transferal to her child. Participation in the study will not decide or in any other way interfere with patients' treatment as prescribed by their physician. Only patients that already have been assigned treatment with prednisolone by their physician will be approached and asked for participation.

7.1. Description of investigational medicinal product(s)

Not applicable.

7.2. Dose and administration

Prednisolone is dosed through several administration routes (oral, inhalation, intramuscular injection, intravenous, ophthalmic). The study will focus on oral administration and will not alter the route of administration, the dosage or the dosage regimen for prednisolone but the participants will continue their treatment as prescribed by their physician.

7.3. Drug accountability and treatment compliance

It will be assumed that the drug prescribed by the doctor is the correct drug. Women taking drugs administered by a pharmacy will self-report their use of medicines in online questionnaires.

Participants self-register the medicines they are using adjacent to sampling of breast milk and blood.

7.4. Randomization and blinding

No randomization or blinding will be performed.

7.5. Concomitant use of other medicinal products and treatments

Not applicable

7.6. Treatment after trial end

Not applicable, the study will not influence choice of treatment or dosage. The patients will continue with their prednisolone treatment, as prescribed by their physician.

8. Methods for measurement of endpoints for clinical efficacy and safety

8.1. Methods for measurement of endpoints for clinical efficacy

The project will only measure pharmacokinetic data of breast milk and blood related to a medicine that is already approved and administered in accordance with standard clinical care outside the protocol of this project. The project will not do any assessment of efficacy related to the treatment itself.

8.2. Methods for measurement of endpoints for clinical safety

For pharmacological safety the project will measure pharmacokinetic data of breast milk and blood related to a medicine that is already approved and administered in accordance with standard clinical procedures for women outside the protocol of this project. Adverse events that may impact on breastfeeding and/or breastmilk, e.g., mastitis, will be documented.

8.2.1. Specification of pharmacological safety parameters

Dosage of infant as Average Daily Infant Dose (ADID) in mg/kg/day will be estimated by using 200 mL/kg/day as a standard for daily milk intake in accordance with FDA guidelines for lactation studies (FDA Clinical Lactation Studies May 2019): “While a 150 mL/kg/day estimated milk intake is a reasonable assumption to estimate daily infant dosage, greater volumes do occur in early infancy and often correlate to the time of most reported infant adverse drug events. Additional consideration should be given to estimates of infant risk based on a 200 mL/kg/day milk intake in early infancy”.

Relative infant dose (RID) will be calculated by dividing infant dosage (ADID) in mg/kg/day with maternal dosage in mg/kg/day, multiplied with 100 in order to get the percentage. It is estimated that a RID < 10% is considered safe for the infant (Bennet PN, Use of the monographs on drugs, Drugs and Human Lactation, 1996:67-74.).

The maternal prednisolone/prednisone milk to plasma ratio will be calculated by dividing drug concentration in the mother’s milk with the drug concentration of the mother’s plasma.

Prednisolone concentration in infant blood will be reported.

Cortisol concentration in infant blood will be reported.

9. Handling of Adverse Events

Only serious adverse events (SAEs) and, for the women, signs of mastitis related to the breast milk sampling will be monitored and documented in a protocol. Other adverse events (AEs) related to blood and milk sampling will not be collected, e.g., pain or bruising at the injection site, as these are expected during sampling procedures.

Because all subjects included in the trial have started the prednisolone treatment before trial enrollment and the treatment is performed according to clinical praxis, no adverse events related to the medicinal product will be collected during the trial and the Sponsor will not perform SUSAR assessments.

9.1. Definitions

9.1.1. Adverse Event (AE)

Adverse Event (AE): Any untoward medical occurrence in a subject to whom a medicinal product is administered and which does not necessarily have a causal relationship with this treatment.

9.1.2. Adverse Reaction (AR)

In the pre-approval clinical experience with a new medicinal product or new use of a medicinal product, and particularly as the therapeutic dose(s) may not be established, all noxious and unintended reactions to the medicinal product related to any dose should be considered an adverse reaction (AR). The phrase “reaction” to a medicinal product means that the causal relationship between the medical product and an adverse event is at least a reasonable possibility, that is the relationship cannot be ruled out.

No investigational medicinal product will be administered during the study. Thus, no ARs will be reported.

9.1.3. Serious Adverse Event (SAE)

Serious adverse event (SAE): Any untoward medical occurrence that at any dose requires inpatient hospitalization or prolongation of existing hospitalisation, results in persistent or significant disability or incapacity, results in a congenital anomaly or birth defect, is life-threatening, or results in death.

Medical and scientific assessment will be made to determine if an event is serious and whether it would prompt reporting in other situations, for example important medical events that may not be directly life-threatening or result in death or hospitalization but may compromise the subject or may require intervention to prevent one of the other results set forth in the definitions above. These should also normally be considered as SAEs.

9.1.4. Suspected Unexpected Serious Adverse Reaction (SUSAR)

SUSAR: A reaction/event that is unexpected, serious, and suspected to be caused by the treatment, i.e. adverse reactions that are not included in the Investigator's Brochure (IB) or SPC.

No investigational medicinal product will be administered during the study. Thus, no SUSARs will be reported.

9.2. Assessment of Adverse Events (AE)

9.2.1. Assessment of seriousness

The investigator is responsible for assessing the seriousness (serious or non-serious). If the adverse event is considered serious, this should be reported as a serious adverse event (SAE) by the investigator to the sponsor. See also section 1.1.9.3.1, Reporting of Serious Adverse Events (SAE).

9.3. Reporting and registration of Adverse Events

9.3.1. Reporting of Serious Adverse Events (SAE)

It is considered highly unlikely that a serious adverse events (SAE) would occur during collection of samples from the participating women and children. The blood samples are drawn at the study site by experienced staff and all subjects are carefully monitored.

If the investigator assesses an event that occurred during sampling as serious, they should contact the sponsor as soon as possible by phone or email (see contact information below). An SAE report should also be filled out. Follow-up information describing the outcome and handling of the SAE is reported as soon as this information is available.

Sponsor representative: Mats Hansson
Email: mats.hansson@crb.uu.se
Phone: 018-4716197

9.3.2. Reporting of Suspected Unexpected Serious Adverse Reactions (SUSAR)

Not applicable.

9.4. Follow-up of Adverse Events

Signs of mastitis occurring in association with breast milk sampling will be monitored and documented at the sampling location and referral be made to an ordinary care unit.

If an SAE occurs during sampling, the subject will be monitored until the SAE is resolved.

9.5. Independent Data Monitoring Committee

Not applicable.

9.6. Annual Safety Report (ASR)

An annual safety report will be submitted in CTIS.

9.7. Procedures in case of emergencies, overdose or pregnancy

Not applicable. See reporting of SAEs 9.3.2.

10. Statistics

In general, summary statistics (n [number of available measurements], arithmetic mean, standard deviation [SD], median, minimum, and maximum) for quantitative variables and frequency tables for qualitative data will be presented. For prednisolone/prednisone concentrations, summary statistics will include geometric mean, geometric coefficient of variation (CV), 95% confidence intervals, arithmetic mean, arithmetic SD, median, minimum, and maximum. Any deviations from this general approach will be outlined in the SAP.

Values that are below the limit of quantification (BLQ) will be set to half the lower limit of quantification (LLOQ) for the calculation of descriptive statistics. Descriptive statistics will be calculated if at least 2/3 of the values are above the LLOQ. If this is not the case, only median, minimum, and maximum will be presented.

The concentration of prednisolone in the breastfeeding child's plasma 3 hr after maternal dose intake including ADID and RID will be summarized descriptively. The concentration of cortisol in the infant blood will also be summarized.

The concentration of prednisolone/prednisone in breast milk at 1h after maternal dose intake, the maternal plasma concentration of prednisolone/prednisone at 1h after maternal dose intake, and the milk to plasma ratio of the mother will be summarized descriptively.

10.1. Analysis population

All subjects included

10.2. Statistical analyses

10.2.1. Statistical methods

In general, summary statistics (n [number of available measurements], arithmetic mean, standard deviation [SD], median, minimum, and maximum) for quantitative variables and frequency tables for qualitative data will be presented. For prednisolone/prednisone concentrations, summary statistics will include geometric mean, geometric coefficient of variation (CV), 95% confidence intervals, arithmetic mean, arithmetic SD, median, minimum, and maximum. Any deviations from this general approach will be outlined in the SAP.

10.2.2. Drop-outs

All data regarding sampling with information on missing or spurious data, e.g., due to effect of other medications, will be recorded at sampling and stored at a secure dataportal (Allvis) at Uppsala University.

10.3. Sample size calculations

No formal sample size calculations have been performed as there are no statistical hypotheses being tested. The planned sample size for this study is 30 subjects. This sample size should provide sufficient data to assess the primary objective of the study.

10.4. Interim analysis

No interim analysis is planned for this study.

11. Quality Control and Quality Assurance

11.1. Quality Assurance and Sponsor oversight

Sampling follows established clinical routines at each site and is performed by specially trained staff. Biobanking and pharmacokinetic analyses follow qualified procedures.

The involved investigators and institutions will permit trial-related monitoring, audits, IRB/IEC review, and regulatory inspections, providing direct access to source data/documents.

Meetings with investigators, biobanking staff and sponsor representatives will be held at regular intervals in order to monitor that sampling, biobanking and analyses follow the protocol. Follow up of each sampling procedure will be made through the filled in report forms and trial specific documentation from each clinical site.

11.2. Monitoring

Sponsor representatives will monitor the trial in order to ensure that the subjects' safety and integrity are satisfied and check that reported data is reliable and of high quality.

Quality control will be attained through monitoring:

- that subjects exist
- that informed consent has been signed prior to execution of any trial-specific actions
- that subjects are included according to the protocol’s inclusion and exclusion criteria
- that the trial’s main parameters and safety reporting are handled correctly

11.3. Source data

Data collected when sampling breast milk

Data collected from mother

Gestational age for most recent birth (self-reported)
Age (self-reported)
Medical condition that motivates treatment with prednisolone (self-reported)
Height (self-reported)
Weight (self-reported)
Any medication(s), and the doses, she is currently using (self-reported)
Any traditional medicinal products , and doses, she is currently using (self-reported)
Dosage of prednisolone (self-reported)
Information on whether the woman can remember missing a dose of prednisolone in the last three days
Time-point for dose intake (0h)
Age of infant (self-reported)
Infant height (self-reported)
Infant weight (self-reported)
Infant medicine use (self-reported)
Any traditional medicinal products the infant is using (self-reported)
Time for last breastfeeding/ bottle feeding of the expressed breast milk
Time for starting breast milk expression (self-reported)
Information whether the woman is exclusively breast-feeding or if the infant is getting supplemental food

Data collected at maternal blood sampling and infant breastfeeding at 1 hr

Data collected by personnel immediately after drawing blood from mother
Time-point for blood sampling
Venipuncture site selection
How blood was drawn (e.g., venipuncture, peripheral venous catheter, port-à-cath)
Tube type used for blood drawing
Any venipuncture equipment used to draw blood
(self-reported)
Time-point for breast milk sampling (reported by health care professional)

Data collected at infant blood sampling at 3h
Time-point for blood sampling
Site selection
How blood was drawn (e.g., back of hand)
Tube type used for blood drawing
Collected data will be pseudonymized and encoded. Data and key to the code will be kept separately. Study participants will be given a Study-ID when enrolled and consented.

Source data, SOPs, master protocols and templates will be stored at Uppsala Biobank. Bioanalytical data (validation, stability, and measured drug concentration values from clinical samples) will be stored at UDOPP.

11.4. Deviations, serious breaches and other reporting obligations

The investigators at each clinical site will, without delay, report to the sponsor any serious breaches and deviations from the trial protocol, ICH-GCP and other regulations that significantly and directly affect, or with high likelihood could affect, the subjects' safety and integrity or the reliability and robustness of the data generated in the trial. The sponsor will assess the suspected serious breach and the consequences of deviations that have occurred, and, without undue delay but no later than 7 days (from knowledge) report these to the Swedish Medical Products Agency via CTIS.

Other unexpected events that may affect the benefit/risk relationship will be reported via CTIS without undue delay, but no later than 15 days after the sponsor becomes aware of the event.

Minor deviations that do not affect subjects' integrity or safety, nor significantly affect the trial's scientific value, are documented in the trial documentation of the principal investigator and the sponsor and appropriate measures will be taken. The deviations will be recorded in the clinical trial report.

11.5. Audits and inspections

Authorized representatives for the sponsor and Competent Authorities (CA) may carry out audits or inspections at the trial site, including source data verification. The investigator will ensure that all source documents are available for audits and inspections.

12. Ethics

12.1. Compliance to the protocol, ICH-GCP and regulations

The trial will be performed in compliance with the EU regulation on low-intervention clinical trials on medicinal products for human use (536/2014), the Declaration of Helsinki, ICH-GCP standards applicable to the trial procedures, and current national regulations governing this clinical trial. This is to ensure the safety and integrity of the trial subjects as well as the quality of the data collected.

12.2. Ethical review of the trial

The final protocol for clinical trials on medicinal products will be approved, as a part of the application for a permit for clinical trials via CTIS, by both the Swedish Ethical Review Authority and the Swedish Medical Products Agency before the trial will be conducted. The authority will be informed via CTIS of any changes in the trial protocol in accordance with current requirements.

12.3. Procedure for obtaining informed consent

The principal investigator at each site will ensure that the subject is given full and adequate oral and written information about the trial, its purpose, any risks and benefits as well as inclusion and exclusion criteria. Subjects will also be informed that they are free to discontinue their participation in the trial at any time without having to provide a reason. Subjects will be given the opportunity to ask questions and be allowed time to consider the provided information.

If the person chooses to participate, both the subject and the investigator shall sign the informed consent form. The women participating in the trial will also ask their partners (the other care giver) to sign). They will bring the signed informed consent form with them to the medical doctor at the sampling center who will sign the form. A copy of the subject information as well as the informed consent form will be provided to the woman. The subject's signed and dated informed consent will be obtained before any trial-specific activity is performed. Each subject who participates in the trial will be identified by a subject number on a subject identification list.

12.4. Data protection

In the information provided to subjects, subjects will be fully informed about how their trial data will be collected, used and disclosed. The content of the informed consent form complies with relevant integrity and data protection legislation. The subject information and the informed consent form will explain how trial data are stored to maintain confidentiality in accordance with national data legislation (see section 14). All information processed by the sponsor will be pseudonymized and coded.

The informed consent form will also explain that for verification of the data, representatives delegated by the sponsor, as well as relevant authorities, may require access to parts of medical records or trial records that are relevant to the trial, including the subject's medical history.

12.5. Insurances

No insurance except the ordinary Patient damage insurance.

13. Substantial changes to the trial

Substantial changes to the signed clinical trial protocol are only possible through approved protocol amendments.

In the event that substantial changes to the protocol which may affect the safety, rights of subjects or the reliability and robustness of data generated need to be implemented during the course of the trial, permission from the relevant authority via application in CTIS will be obtained before implementing the change. This includes the addition of a new trial site or a change of the principal investigator at the trial site.

Non-substantial amendments are entered into the CTIS in the next substantial amendment application concerning the same part. If the non-substantial change is relevant to the Authority's oversight (e.g. contact details), the CTIS will be updated on an ongoing basis.

14. Collection, handling, and archiving of data

Sampling details and health-related details will be collected using a paper questionnaire and then registered in the program REDCAP (Research Electronic Data Capture). Informed consent forms are sent by registered mail to Uppsala University and stored in a locked cabinet only accessible by authorised personnel. Data will be uploaded in an encrypted format which then will be hosted at a locked and secure server of Uppsala University (Dataportal Allvis) and Region Uppsala in accordance with an established process approved by the Swedish Ethical Review Authority. Collected data will be pseudonymized and encoded. Data and key to the code will be kept separately. Study participants will be given a Study-ID when enrolled and consented.

The complete Trial Master File with essential documents will be archived for at least 25 years. Source data in the medical records system are stored and archived in accordance with national regulations.

14.1. Case Report Form

A Case Report Form (CRF) is used for data collection. It includes three separate paper forms filled in at sampling (See attachments). The investigator will ensure that data is registered and any corrections in the CRF are made as stated in the clinical trial protocol and in accordance with the instructions. The investigator will ensure that the registered data is correct, complete, and that reporting takes place according to the timelines that have been predefined and agreed. The principal investigator signs the completed forms. A copy of the completed forms will be archived at the site.

15. Notification of trial completion, reporting, and publication

End of the trial is reported in CTIS at the latest 15 days after completion.

Within one year of trial completion, a clinical study report is completed, and a summary of the clinical trial results will be reported in CTIS, including a summary for lay people.

Scientific publications based on the study results will adhere to Vancouver guidelines.

16. References

- Bermas BL. Lactation and Management of Postpartum Disease. Rheum Dis Clin N Am 43 (2017) 249–262.
- Constantinescu S, Pai A, Coscia LA, et al. Breast-feeding after transplantation. Best Pract Res Clin Obstet Gynaecol 2014;28:1163–73.
- Greenberger PA, Odeh YK, Frederiksen MC, et al. Pharmacokinetics of prednisolone transfer to breast milk. Clin Pharmacol Ther. 1993;53:324–8.

- Katz FH, Duncan BR. Entry of prednisone into human milk [letter]. N Engl J Med 1975;293:1154.
- Ost L, Wettrell G, Bjorkhem I, et al. Prednisolone excretion in human milk. J Pediatr 1985;106:1008–11.

17. Attachments

Frågeformulär 1-3

Frågeformulär 1 (fylls i av deltagare vid provtillfället för bröstmjölk)

Studie: Läkemedelsbehandling med Prednisolon i samband med amning

Deltagares studie-ID:

Datum: _____/_____/_______ (t. ex. 20/01/2012)

Tid: __________

Instruktion:

Detta frågeformulär syftar till att samla in information som skall användas vid analys av dina prover. Informationen kommer att behandlas så att inte obehöriga kan ta del av den. ***Detta frågeformulär fylls i efter bröstmjölksprovet.***

Information om dig

1. **Hur gammal är du?** ______ år
2. **Hur lång är du?** ______ cm
3. **Hur mycket väger du?** _____kg
4. **För vilken diagnos tar du Prednisolon?**

[Fritext:] ____

5. **Använder du några andra mediciner?**

☐ Nej ☐ Ja

Om ja, ange namn: ____

6. **Använder du något/några naturläkemedel?**

☐ Nej ☐ Ja

Om ja, ange namn: ____

7. **Vilken dos av Prednisolon tar du? (till exempel 1 tablett á 20 mg per dag)?**

[Fritext:] ____

8. Ange när du tog din senaste dos av Prednisolon:

Datum: _____/_____/_______ (t. ex. 20/01/2012)

Tid: ___________

9. Ange om du kan minnas att du har missat en dos under de senaste tre dagarna:

☐ Jag har inte missat en dos under den tiden

☐ Jag missade en dos igår

☐ Jag har missat en dos i förrgår

☐ Jag är osäker

Information om ditt barn

10. Hur gammalt är ditt barn? [Fritext:] ______________________

11. I vilken graviditetsvecka föddes ditt barn? I vecka_____ och dag _____

12. Hur lång är ditt barn? _____ cm

13. Vad väger ditt barn? _____ g

14. Vilken mat får ditt barn?

☐ Mitt barn ammas enbart

☐ Mitt barn delammas och får bröstmjölksersättning

15. Ges ditt barn för närvarande läkemedel/örter/vitaminer/mineraler?

☐ Nej ☐ Ja

Om ja, ange namn på preparatet: ____

Information om provtagning av bröstmjölk

16. Ange när du senast ammade/gav ditt barn urpumpad bröstmjölk:

Datum: _____/_____/_______ (t. ex. 20/01/2012)

Tid: ___________

17. Vid vilken tidpunkt började du pumpa ur bröstmjölk?

Datum: _____/_____/_______ (t. ex. 20/01/2012)

Tid: ___________

Frågeformulär 2 (fylls i av personal efter blodprov)

Studie: Läkemedelsbehandling med Prednisolon i samband med amning

Deltagares studie-ID:

Datum: _____/_____/_______ (t. ex. 20/01/2012)

Tid: __________

Instruktion:

Detta frågeformulär syftar till att samla in information som skall användas vid analys av deltagarens prover. Informationen kommer att behandlas så att inte obehöriga kan ta del av den. ***Detta frågeformulär fylls i efter blodprovstagningstillfället.***

Information om blodprovstagning

1. Ange datum och tidpunkt för blodprovtagning:

Datum: _____/_____/_______ (t. ex. 20/01/2012)

Tid: __________

2. Ange vart blodprovet togs:

☐ vänster arm ☐ höger arm ☐ bägge sidor ☐annan lokalisation

3. Hur togs blodprovet:

☐ Genom venpunktion

☐ Från en perifer venkateter (PVK)

☐ Från en port-a-cath (subkutan venport, SVP)

☐ Från en PICC-line

☐ Från en annan typ av kateter: ____

4. Ange vilken typ av provrör som användes:

____

5. Ange vilken typ av utrustning som användes för venöst blodprov:

____

6. Ange datum och tidpunkt för lämnande av bröstmjölksprov:

Datum: _____/_____/_______ (t. ex. 20/01/2012)

Tid: __________

7. Övrig information:

[Fritext:] ____

____

____

____

Frågeformulär 3 (fylls i av personal efter blodprov på barnet)

Studie: Läkemedelsbehandling med Prednisolon i samband med amning

Deltagares studie-ID:

Datum: _____/_____/_______ (t. ex. 20/01/2012)

Tid: __________

Instruktion:

Detta frågeformulär syftar till att samla in information som skall användas vid analys av blodprovet som tagits på deltagarens barn. Informationen kommer att behandlas så att inte obehöriga kan ta del av den. ***Detta frågeformulär skall fyllas i en gång, efter att blodprov tagits på barnet.***

Information om blodprovstagning

18) Ange datum och tidpunkt för blodprovtagning av barnet:

Datum: _____/_____/_______ (t. ex. 20/01/2012)

Tid: __________

19) Ange vart blodprovet togs (t. ex häl, handrygg osv):

____

20) Ange vilken typ av provrör som användes:

____

21) Övrig information:

[Fritext:] ____

____

____

____
