# Supplementary material for: Determination of prednisolone concentration in human breast milk and plasma of breastfed infants: study protocol of a Swedish multicentre low-intervention clinical trial
Source: BMJ Open. 2025 May 22;15(5):e097898. doi: 10.1136/bmjopen-2024-097898 (PMC12097052; doi:10.1136/bmjopen-2024-097898)
Supplement: Supplementary file 2 [file bmjopen-2024-097898.reviewer_comments.pdf]

PEER REVIEW HISTORY

BMJ Open publishes all reviews undertaken for accepted manuscripts. Reviewers are asked to complete a checklist review form and are provided with free text boxes to elaborate on their assessment. These free text comments are reproduced below.

ARTICLE DETAILS

Title (Provisional)

Determination of prednisolone concentration in human breast milk and plasma of breastfed infants – study protocol of a Swedish multicenter low-intervention clinical trial.

Authors

Svedenkrans, Jenny; Hellgren, Karin; Backlund, Karin; Perrin, Nina; Timby, Erika; Nilsson, Karin; Drevin, Jennifer; Sundell, Erica; Shaughnessy, Laura; Hansson, Mats

---

VERSION 1 - REVIEW

**Reviewer**	**1**
**Name**	**D. Roghair, Robert**
**Affiliation**	**Stead Family Department of Pediatrics, Carver College of Medicine, University of Iowa**
**Date**	**14-Feb-2025**
**COI**	**None**

This is a very well written and well-planned ongoing protocol. There are a few points that could be worth clarifying in the final publication of the results. 1. How was the sample size determined? It is stated that no power calculation was performed, which is fine, but was the sample then based on the anticipated recruitment through 2025 or some other consideration? 2. There is no rationale provided for collecting serum cortisol levels. 3. Is there a plan to exclude infants in the unlikely event that they are themselves receiving prednisolone? 4. The investigations are timely with this recent article now increasing parental concern about prednisolone exposure (Laugesen et al. JAMA Network Open 2025 PMID: 39752154), and while that citation deals with prenatal and not breast milk exposure, it might still be worth citing it in the final publication of this study's results.

**Reviewer**	**2**
**Name**	**Datta, Palika**
**Affiliation**	**Texas Tech University Health Science Center**
**Date**	**19-Feb-2025**

COI None

The study protocol is well-written; however, the methodology for the analysis and quantification of drugs in milk and plasma samples lacks clarity. To ensure transparency and reproducibility, please provide a detailed description of the analytical techniques used or reference a specific, validated method that is being followed.

Reviewer 3

Name Annaert, Pieter

Affiliation University Hospital Leuven

Date 14-Mar-2025

COI I am currently also involved in the conduct of clinical lactation studies as a co-investigator. In addition, I am involved in designing, supervising and evaluating bioanalysis methods for milk and plasma, as applied in this research.

This manuscript reports the rationale and contents of a protocol for a clinical lactation study applied to prednisolone. Although the title generates the impression that data have been collected and will be reported in the manuscript, this is not the case. This should probably be indicated in the title by mentioning 'Protocol for'?

The study addresses a high unmet medical need in terms of information currently lacking regarding transfer of prednisolone and other drugs in human breastmilk.

Several improvements can be made, for instance in terms of sampling time point and/or justification should be added why sampling is not done at trough.

Specific comments to be addressed are as follows:

1. Abstract: since medicines are reaching the milk at the blood-milk barrier and not (directly) leaving the body during the secretion process, it is more correct to refer to drug milk secretion rather than drug milk excretion.

2. The 1h and 2h time points are a bit arbitrarily chosen. As these time points are possibly close to the peak in some patients but more distant in other patients (because of slow absorption; what is the effect of food?), high variability may be expected. Did the authors consider taking trough samples? This would have been more useful given the fact that the study is conducted at steady state as mentioned further in the protocol.

3. The first strength of the study: "The main strength of the study is that the amount of prednisolone transferred through the breast milk will not just be estimated, but measured.", reads a bit ambiguous. On one hand just 1 time point is collected (making the average

concentration an estimate), while on the other hand the act of measuring is obvious from the title of the study.

4. Are there also EMA recommendations for lactation studies?

5. Please specify the type and objective of the follow-up studies.

6. Can a rationale be added as to why exactly prednisolone was identified from 12 drugs that met the criteria? It would also be interesting for the reader to know the 12 drugs.

7. In the “what is already known” paragraph, RID’s of the published studies can at least be estimated, since the % of the maternal dose is known. Typical bodyweights can be used for the calculation.

8. Information on the physicochemistry of prednisolone in relation to the anticipated propensity of milk partitioning is lacking.

9. Exact definitions and/or equations of ADID and RID are lacking on p.4 under ‘The demonstration study’

10. On p.5, reference is made to EMA and FDA guidelines, but no specific guidelines are cited.

11. Please give examples of how genetics could potentially affect drug transfer.

12. The samples are frozen and shipped on ice. Is this dry ice? If yes, this should be mentioned.

13. What is the difference between ADID and DID? It should at least be specified what the ‘average’ refers to? Is this across different patients, or rather based on sampling within the same patient. Note that for an average concentration in plasma or milk, the AUC divided by dosing interval tau should be used.

14. Is the RID <10% threshold still commonly used? Given some controverse around it, nuance can be added in the text, considering intrinsic toxicity of the drug in question.

15. Details about the performance and linearity including LLOQ of the method are missing.

16. The preliminary results section refers to Oct 1st, 2024. Are recent updates available?

17. Reference to some other lactation protocols, including UmbrelLACT (also under IMI ConcePTION) is lacking (PMID: 38599799).

18. In the protocol, the exclusion criteria state “…multiple birth.” This may be misinterpreted.

19. In section 8.2.1 of the protocol, the ADID calculation is lacking. Only a volume is mentioned.

20. The protocol should mention the limitations associated with determining milk to plasma ratio based on single time points.

21. The statistics section of the protocol contains repetition.

Minor/other:

- The current title is not grammatically correct: the part "…and the plasma of breastfeeding children" is disconnected from the first part of the sentence.

- Under strengths and limitations: "A limitation of the study is that it is only conducted in Sweden, thereby limiting the relevance for other countries where prescription patterns may be variable.": replace 'variable' by 'different'.

- 'Transfer' instead of 'transferal'

VERSION 1 - AUTHOR RESPONSE

Reviewer: 1
Dr. Robert D. Roghair, Stead Family Department of Pediatrics, Carver College of Medicine, University of Iowa
Comments to the Author:
This is a very well written and well-planned ongoing protocol. There are a few points that could be worth clarifying in the final publication of the results.

1. How was the sample size determined? It is stated that no power calculation was performed, which is fine, but was the sample then based on the anticipated recruitment through 2025 or some other consideration?

The sample size was chosen in order to have sufficient data for a clear conclusion for the primary endpoint of prednisolone concentration in the breastfeeding child. Sample size considerations included pharmacokinetic and pharmacodynamic variability for the drug under study, and the variability in lactation physiology including inter- and intra-subject variability for mother and the breast-fed child. The considerations were made in accordance with the FDA lactation studies guidance from 2019 and 2005 where 6-8 subjects were considered to suffice in milk-only studies. Furthermore, the choice to analyze peak concentrations and to administer all doses together with food, were considered to reduce effects of intra- and intersubject variability. A sample size of 25 pairs were considered to be sufficient. Given that approximations had to be used, the sample size was increased to 30. A short explanation has been added to the text.

Commented [JS1]: Jenny- this text is from the earlier version of the FDA lactation guidance that I noted to you. I hope it is sufficient.

Commented [JS2R1]: The sample size was chosen in order to have sufficient data for a clear conclusion for the primary endpoint of prednisolone concentration in the breastfeeding child. Sample size considerations included PK and PD variability for the drug under study, and the variability in lactation physiology. Inter- and intra-subject variability for mother and the breast-fed child.

2. There is no rationale provided for collecting serum cortisol levels.
Thank you for highlighting this. We have added a rationale for this in the introduction. The serum cortisol is collected to get a measurement of clinical effects in the infant.

3. Is there a plan to exclude infants in the unlikely event that they are themselves receiving prednisolone?
We didn't think about this unlikely event when designing the study. However, no infant treated with prednisolone will be included in the study. This has been added to the methods section.

4. The investigations are timely with this recent article now increasing parental concern about prednisolone exposure (Laugesen et al. JAMA Network Open 2025 PMID:

39752154), and while that citation deals with prenatal and not breast milk exposure, it might still be worth citing it in the final publication of this study's results.

Thank you for this comment. We agree that that concerns about prenatal exposure are important also when studying secretion to breastmilk.

Reviewer: 2
Dr. Palika Datta, Texas Tech University Health Science Center
Comments to the Author:
The study protocol is well-written; however, the methodology for the analysis and quantification of drugs in milk and plasma samples lacks clarity. To ensure transparency and reproducibility, please provide a detailed description of the analytical techniques used or reference a specific, validated method that is being followed.

Thank you for this comment. We have added information about analytical techniques.

Commented [JS3]: Double-check text before submission

Reviewer: 3
Dr. Pieter Annaert, University Hospital Leuven
Comments to the Author:
This manuscript reports the rationale and contents of a protocol for a clinical lactation study applied to prednisolone. Although the title generates the impression that data have been collected and will be reported in the manuscript, this is not the case. This should probably be indicated in the title by mentioning 'Protocol for'?

Thank you for this important comment. We have changed the title of the paper to clarify that this is a protocol.

The study addresses a high unmet medical need in terms of information currently lacking regarding transfer of prednisolone and other drugs in human breastmilk.
Several improvements can be made, for instance in terms of sampling time point and/or justification should be added why sampling is not done at trough.

Thank for this comment. Please see specific justifications below.

Specific comments to be addressed are as follows:
1. Abstract: since medicines are reaching the milk at the blood-milk barrier and not (directly) leaving the body during the secretion process, it is more correct to refer to drug milk secretion rather than drug milk excretion.

Thank you for highlighting this. We agree with your comment and have changed "excretion" to "secretion" throughout the paper.

2. The 1h and 2h time points are a bit arbitrarily chosen. As these time points are possibly close to the peak in some patients but more distant in other patients (because of slow absorption; what is the effect of food?), high variability may be expected. Did the authors consider taking trough samples? This would have been more useful given the fact that the study is conducted at steady state as mentioned further in the protocol.

Thank you for highlighting this dilemma. We have chosen to aim for peak levels in plasma and milk, as demonstrated by Ost et al (Prednisolone excretion in human milk, J Ped, 1985). As prednisolone levels have been shown to be very low in milk after 4-6 hours, a design aiming for trough may have resulted in unmeasurable milk levels. Ost et al demonstrated peak plasma levels of prednisolone at 1 hour after administration, and we extrapolated this to be valid also for the infants. We didn't deem investigation of individual pharmacokinetics as feasible as it would have needed more sampling of the study participants. All women take their dose at the study visit and are given something to eat at the same time. This is done to harmonize pharmacokinetics between patients as much as possible, but individual drug uptake is a limitation to the study. We have chosen to include as many as 30 women-infant pairs to be able to draw a robust conclusion.

We have developed this discussion in the text as well.

3. The first strength of the study: "The main strength of the study is that the amount of prednisolone transferred through the breast milk will not just be estimated, but measured.", reads a bit ambiguous. On one hand just 1 time point is collected (making the average concentration an estimate), while on the other hand the act of measuring is obvious from the title of the study.

Thank you for this comment. We consider it to be a strength to measure prednisolone concentration in the plasma of the neonate to describe actual transfer. In previous studies, transfer to the infant was estimated using milk concentrations only. The section "strengths…" have been reformulated to clarify.

We do agree that the average prednisolone concentration in milk and the infant's plasma over the day will be an estimate. We have clarified this in the text.

4. Are there also EMA recommendations for lactation studies?

We consider the EMA recommendations to perform lactation studies as such recommendations. Even though these are briefer, they highlight the importance of lactation studies for all drugs. The reference has been added in the paper.

5. Please specify the type and objective of the follow-up studies.

Future studies have not yet been defined. The biobanking will allow for us to design studies as new research questions arise. The term "follow-up" may not be the most suitable in this context, and the section has been reformulated.

6. Can a rationale be added as to why exactly prednisolone was identified from 12 drugs that met the criteria? It would also be interesting for the reader to know the 12 drugs.

Thank you for highlighting this. A rationale for why prednisolone was chosen has been added to the text.

7. In the "what is already known" paragraph, RID's of the published studies can at least be

Commented [JS4]: Discuss with Laura, Find best refernce.

Commented [LS5R4]: CHMP: GUIDELINE ON RISK ASSESSMENT OF MEDICINAL PRODUCTS ON HUMAN REPRODUCTION AND LACTATION: FROM DATA TO LABELLING, Draft July 2008

Commented [LS6R4]: From this GL: Information on the risks following exposure to the medicinal product during lactation should be provided as well as recommendations on the management of risk in clinical practice.

estimated, since the % of the maternal dose is known. Typical bodyweights can be used for the calculation.

We have added a span of RIDs depending on different assumptions and also refer to the sources of these calculations.

8. Information on the physicochemistry of prednisolone in relation to the anticipated propensity of milk partitioning is lacking.

Commented [JS7]: We have interpreted this as the section below. Please comment if you have other thoughts about this question:

Prednisolone is a small molecule (340 Da) that is neutral in pH in has low solubility in water and high solubility in fat. Small molecules usually pass easily into breast milk which would indicate a higher transfer. With high fat solubility it is expected that higher concentrations are found in hindmilk. The dose-dependent protein binding in plasma is likely the most important predictor of transfer into breast milk. By using a full portion of breast milk, the difference between hind milk and fore milk is considered to be unimportant.

We have added a section about this in the "what is known" section.

9. Exact definitions and/or equations of ADID and RID are lacking on p.4 under 'The demonstration study'

The definitions have been specified and are available under the section "measures". In concordance with another reviewer's valuable comment, we have chosen to use DID instead of ADID.

10. On p.5, reference is made to EMA and FDA guidelines, but no specific guidelines are cited.

Thank you for noticing this. The citations have been added.

11. Please give examples of how genetics could potentially affect drug transfer.

Genetic difference may affect pharmacokinetics such as time to peak plasma levels, distribution, level of protein binding, elimination as well as composition of breastmilk. Furthermore, the infants drug uptake may affect the transfer to the infants' plasma. This has been added to the text.

12. The samples are frozen and shipped on ice. Is this dry ice? If yes, this should be mentioned.

The samples are shipped on dry ice to the Biobank after aliquoting at the local lab. This has been clarified in the text.

13. What is the difference between ADID and DID? It should at least be specified what the 'average' refers to? Is this across different patients, or rather based on sampling within the same patient. Note that for an average concentration in plasma or milk, the AUC divided by dosing interval tau should be used.

Thank you for this very important comment. We agree that the use of ADID is not suitable for the study design as only one measurement per patient is done. According to this we will not use ADID but DID (daily infant dose) instead. We also acknowledge that the calculation of DID will be affected by the single measurement. We have chosen to prioritize measurement in the infant's plasma in this study as we consider that to give more interesting new information than a milk-only study with several sampling time points.

14. Is the RID <10% threshold still commonly used? Given some controverse around it, nuance can be added in the text, considering intrinsic toxicity of the drug in question.

Thank you for highlighting this. We have added some nuances around this threshold in the text. And we agree that the limit is a bit arbitrary but find it to be commonly used.

15. Details about the performance and linearity including LLOQ of the method are missing.

This will be added.

Commented [JS8]: I need help with this one

16. The preliminary results section refers to Oct 1st, 2024. Are recent updates available?

The preliminary results have been updated in this version to the 1st of April 2025.

17. Reference to some other lactation protocols, including UmbrelLACT (also under IMI ConcePTION) is lacking (PMID: 38599799).

Thank you for this suggestion. Reference to other lactation protocol has been added to the paper.

18. In the protocol, the exclusion criteria state "...multiple birth." This may be misinterpreted.

The expression "multiple births" is established in newborn medicine to express twins, triplets or more. Acknowledging that all readers are not familiar with this nomenclature, we have clarified in the text to avoid misinterpretation.

19. In section 8.2.1 of the protocol, the ADID calculation is lacking. Only a volume is mentioned.

Thank you for pointing this out. We have clarified the equation in the article. Please se comment in question 13 as well.

20. The protocol should mention the limitations associated with determining milk to plasma ratio based on single time points.

Thank you, this is an important limitation. We have added this limitation to the text.

21. The statistics section of the protocol contains repetition.
Thank for noticing this. This will be corrected in the next version of the protocol.

Commented [JS9]: Till protokoll

Minor/other:
- The current title is not grammatically correct: the part "...and the plasma of breastfeeding children" is disconnected from the first part of the sentence.

Thank you for noticing this. The title has been changed to clarify that this is a protocol.

- Under strengths and limitations: “A limitation of the study is that it is only conducted in Sweden, thereby limiting the relevance for other countries where prescription patterns may be variable.”: replace ‘variable’ by ‘different’.

Thank you. The wording has been changed accordingly.

- ‘Transfer’ instead of ‘transferal’
Thank you. The wording has been changed accordingly.

Reviewer: 1 competing interests.: Not applicable

Reviewer: 2 competing interests.: Not applicable

Reviewer: 3 competing interests.: I am currently also involved in the conduct of clinical lactation studies as a co-investigator. In addition, I am involved in designing, supervising and evaluating bioanalysis methods for milk and plasma, as applied in this research.
